# Habitat Specialization in Tropical Continental Shelf Demersal Fish Assemblages

**DOI:** 10.1371/journal.pone.0039634

**Published:** 2012-06-25

**Authors:** Ben M. Fitzpatrick, Euan S. Harvey, Andrew J. Heyward, Emily J. Twiggs, Jamie Colquhoun

**Affiliations:** 1 The Oceans Institute and School of Plant Biology, Faculty of Natural and Agricultural Science, The University of Western Australia, Perth, Western Australia, Australia; 2 Oceanwise Environmental Scientists, Perth, Western Australia, Australia; 3 Australian Institute of Marine Science, The Oceans Institute, Perth, Western Australia, Australia; 4 Department of Applied Geology, Curtin University of Technology, Perth, Western Australia, Australia; Swansea University, United Kingdom

## Abstract

The implications of shallow water impacts such as fishing and climate change on fish assemblages are generally considered in isolation from the distribution and abundance of these fish assemblages in adjacent deeper waters. We investigate the abundance and length of demersal fish assemblages across a section of tropical continental shelf at Ningaloo Reef, Western Australia, to identify fish and fish habitat relationships across steep gradients in depth and in different benthic habitat types. The assemblage composition of demersal fish were assessed from baited remote underwater stereo-video samples (n = 304) collected from 16 depth and habitat combinations. Samples were collected across a depth range poorly represented in the literature from the fringing reef lagoon (1–10 m depth), down the fore reef slope to the reef base (10–30 m depth) then across the adjacent continental shelf (30–110 m depth). Multivariate analyses showed that there were distinctive fish assemblages and different sized fish were associated with each habitat/depth category. Species richness, MaxN and diversity declined with depth, while average length and trophic level increased. The assemblage structure, diversity, size and trophic structure of demersal fishes changes from shallow inshore habitats to deeper water habitats. More habitat specialists (unique species per habitat/depth category) were associated with the reef slope and reef base than other habitats, but offshore sponge-dominated habitats and inshore coral-dominated reef also supported unique species. This suggests that marine protected areas in shallow coral-dominated reef habitats may not adequately protect those species whose depth distribution extends beyond shallow habitats, or other significant elements of demersal fish biodiversity. The ontogenetic habitat partitioning which is characteristic of many species, suggests that to maintain entire species life histories it is necessary to protect corridors of connected habitats through which fish can migrate.

## Introduction

Susceptibility of marine organisms to anthropogenic impacts and natural perturbations depend, in part, upon the degree of habitat specialization of fishes, which can be vastly different between closely related species and between different life history stages of the same species [1]. Therefore, an understanding of habitat usage and the requirements of fish at various life stages will aid predictions about how fish distributions might respond to pressures such as climate change, over fishing and pollution [1]. An understanding of habitat usage will also facilitate management of essential fish habitat by enabling managers to assess the representation of habitats within current marine protected areas (MPA's) [2]. Shallow water marine environments are being increasingly exposed to such impacts which threaten the overall maintenance of fish diversity in coral reefs [3], [4]. Coral bleaching on coral reefs associated with increasing sea surface temperatures, is directly affecting the distribution and abundance of fishes, particularly those which are linked to certain coral reef habitats such as some *Pomacentridae* and *Chaetodontidae* species [3], [5], [6]. The ability of fishes to respond to these impacts through range shifts with latitude and depth will likely be influenced by the degree of habitat specialization of the fish [7], [8].

It has been shown that specific fish and benthic habitat associations exist, and that these habitat associations can change throughout fishes life histories [2], [3]. More detailed information on fish and fish-habitat relationships will help develop more robust species distribution models [9], [10]. This will help inform management decisions, such as the design of MPA's to protect entire life history of species and further assist fisheries and conservation planning and management. Two parameters known to explain a large proportion of variability in fish assemblages are depth and habitat, yet very few studies of fish assemblages encompass both shallow and deeper continental shelf habitats [11].

The majority of fish assemblage assessments on coral reefs are limited to 30 m, yet it is becoming increasingly evident that the depth range of many species normally associated with shallow water can extend well below this [8], [12], [13], [14], [15]. There is limited knowledge of the abundance and length distributions of non-commercially important species across continental shelf habitats between 30 and 100 m. Many studies assessing the structure of continental shelf fish assemblages have used trawls to collect data [16]. Trawl surveys are often constrained to low relief habitats due to the danger of snaring gear on rocky outcrops. Additionally, trawling is a coarse sampling tool that is not suitable for discriminating fine scale fish-habitat associations. These constraints often result in the shallow waters of the continental shelf being infrequently sampled and could explain why increasing patterns of diversity with water depth have been reported [16], [17]. Increasing displacement of fisheries effort to the continental shelves following the depletion of shallower water stocks [18], [19], means that developing baselines on the distribution of target species, as well as overall fish assemblage structure in these habitats, is important [20], [21].

**Figure 1:**
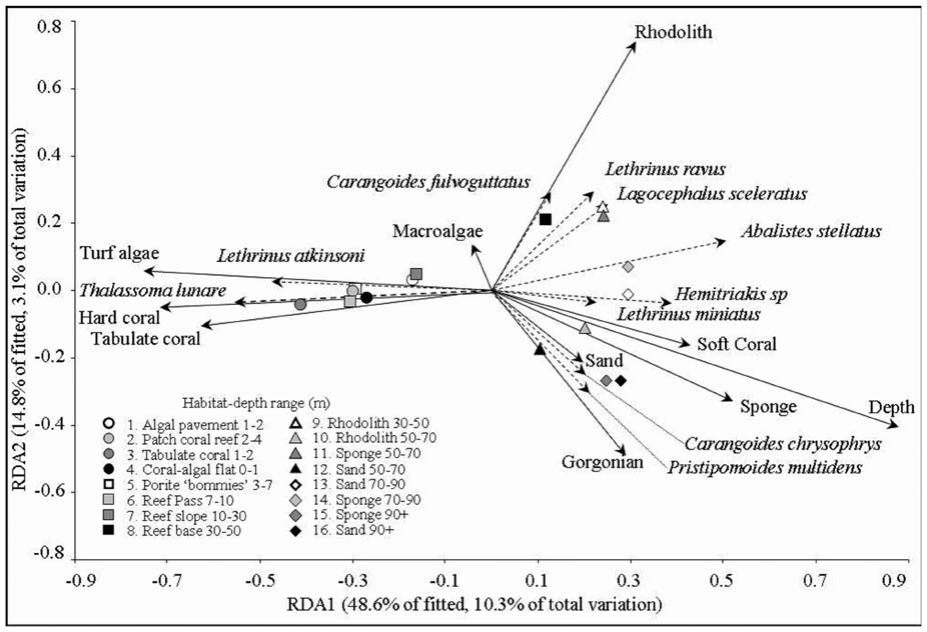
Redundancy analysis biplot representing spearman rank correlations for fish species, habitat variables and centroids of modified Gower log 10 fish assemblage resemblance matrix sampled from each of 16 habitat depth categories. Some of the fish species that contribute greatest similarity between stereo-BRUV replicates from within zones and percent cover of habitat variables correlated to overall assemblage structure are indicated.

**Table 1:** Displaying results of distance based linear model using forward selection and 4999 permutations.

SEQUENTIAL TESTS					Prop	Cumul.
Variable (% cover)	AIC	SS(trace)	Pseudo-F	P	%	%
Depth	−125.63	18.48	28.12	>0.001	8.5	8.5
Rhodolith % cover	−134.23	6.80	10.68	>0.001	3.1	11.7
Hard Coral % cover	−137.80	3.48	5.55	>0.001	1.6	13.3
Sponge % cover	−139.03	1.99	3.19	>0.001	0.9	14.2
Coralline algae % cover	−140.16	1.91	3.08	>0.001	0.9	15.1
Gorgonian % cover	−141.06	1.75	2.85	>0.001	0.8	15.9
Seawhip % cover	−142.09	1.81	2.97	>0.001	0.8	16.7
Branching coral % cover	−143.15	1.81	2.98	>0.001	0.8	17.5
Macroalgae % cover	−143.85	1.58	2.62	>0.001	0.7	18.3
Sand % cover	−144.73	1.67	2.78	>0.001	0.8	19.0
Turf Algae % cover	−145.04	1.33	2.23	>0.001	0.6	19.6
Tabulate coral % cover	−145.33	1.31	2.20	>0.001	0.6	20.2
Seagrass % cover	−145.46	1.21	2.04	0.004	0.6	20.8

The AIC selection criteria was recorded with proportion of variability in modified gower log 10 similarity matrix of species MaxN explained by individual environmental variables noted individually and cumulatively. These 13 out of 19 variables were the best combination of predictor variables identified and accounted for a total of 20.8% variation.

The aims of this study were to 1) investigate the structure of the demersal fish assemblages from the inner lagoon to outer shelf across a range of benthic habitats, 2) determine whether the abundance and length of demersal fishes differed across the shelf, 3) identify fish and fish-habitat relationships for key species and families of interest to assess whether existing shallow water MPA’s are representing the fish assemblages of adjacent deeper habitats. Baited stereo remote underwater video (stereo-BRUVS) were used because they are non-destructive, but not constrained to shallow depths like SCUBA divers and utilize well established design, calibration and measurement procedures [22], [23], [24], [25]). Similarly, they are not limited in the range of benthic habitats that can be sampled like destructive sampling techniques like trawling can be. They have been shown to sample a broad array of the fish compared to other sampling techniques however like all other non-destructive sampling techniques they do not sample the entire assemblage [26], [27], [28], [29]. They are also cost and time effective and so a large number of replicates can be collected following robust experimental designs with strong statistical power [30]. Stereo-BRUVS do not provide an absolute measure of fish abundance rather the maximum number of individuals (MaxN) of a particular species that can be seen in the field of view of the camera at any one time is derived.

**Table 2:** PERMANOVA results displaying the significance of interactions between overall MaxN, species MaxN, species, genus, family, order and class richness, overall length and trophic level and Shannon diversity; and depth (10 degrees of freedom), habitat (11 degrees of freedom) and depth/habitat terms, using 4999 permutations.

	Source	df	MS	Pseudo-F	P(perm)
Overall MaxN	Habitat	4	17617	5.2737	**>0.001**
Univariate	Depth	3	728.74	0.21815	0.87
Euclidean	HaxDe*	2	77.213	2.31E-02	0.968
distance	Res	288	3340.5		
	Total	303			
Overall length	Habitat	4	83558	7.4916	**>0.001**
Univariate	Depth	3	36184	3.2441	0.028
Euclidean	HaxDe*	2	26577	2.3828	0.096
distance	Res	257	11154		
	Total	272			
Species MaxN	Habitat	5	1.991	3.4287	**>0.001**
multivariate	Depth	4	1.6249	2.7981	**>0.001**
mod. Gower	HaxDe*	2	0.91565	1.5768	**>0.001**
log 10	Res	287	0.58069		
	Total	303			
Species richness	Habitat	4	1218.3	23.19	**>0.001**
Univariate	Depth	3	23.453	0.44643	0.719
Euclidean	HaxDe*	2	8.5108	0.16201	0.856
distance	Res	288	52.534		
	Total	303			
Overall trophic level	Habitat	4	0.56659	15.586	**>0.001**
Univariate	Depth	3	5.45E-02	1.4994	0.202
Euclidean	HaxDe*	2	7.76E-02	2.1355	0.128
distance	Res	288	3.64E-02		
	Total	303			
Shannon diversity	Habitat	4	6.2634	23.147	**>0.001**
Univariate	Depth	3	0.37354	1.3804	0.249
Euclidean	HaxDe*	2	0.2483	0.91759	0.395
distance	Res	288	0.2706		
	Total	303			
Genus richness	Habitat	4	2.45E+09	21.177	**>0.001**
multivariate	Depth	3	1.04E+09	9.0143	**>0.001**
mod. Gower	HaxDe*	2	2.82E+08	2.4318	0.115
log 10	Res	288	1.16E+08		
	Total	303			
Family richness	Habitat	4	2.2515	8.2225	**>0.001**
multivariate	Depth	3	0.91218	3.3313	**>0.001**
mod. Gower	HaxDe*	2	0.55837	2.0392	0.062
log 10	Res	288	0.27382		
	Total	303			
Order richness	Habitat	4	1.6555	8.6112	**>0.001**
multivariate	Depth	3	0.51102	2.6581	**0.002**
mod. Gower	HaxDe*	2	0.2629	1.3675	0.198
log 10	Res	288	0.19225		
	Total	303			
Class richness	Habitat	4	1.7741	14.213	**>0.001**
multivariate	Depth	3	0.2827	2.2648	0.055
mod. Gower	HaxDe*	2	0.21747	1.7423	0.157
log 10	Res	288	0.12482		
	Total	303			

Figures in bold indicate significant results. * Term has one or more empty cell.

**Figure 2:**
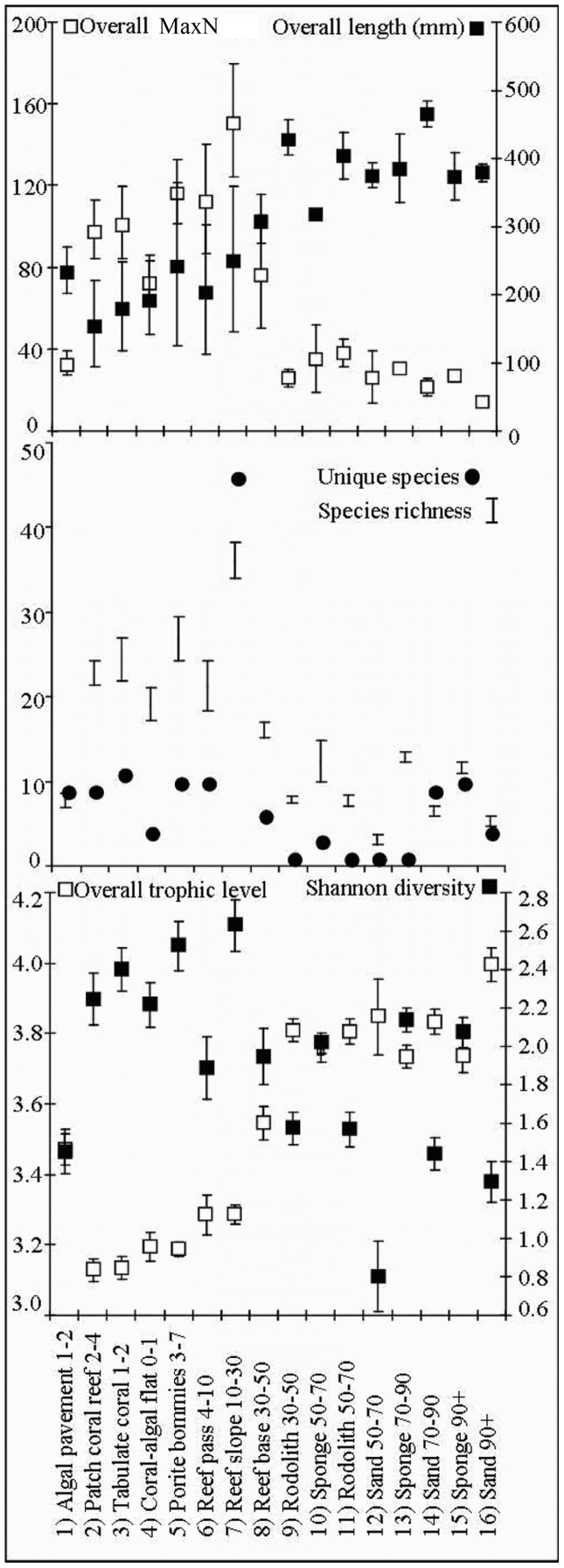
Average overall MaxN, length and trophic level, Shannon diversity, number of unique species and average species richness per stereo-BRUV replicate within each habitat zone. Axes titles are situated at the head of each axis with the units (where appropriate) displayed in brackets. Unique species and species richness are represented on the same axis as whole numbers. Overall trophic level and Shannon diversity are relative measures and do not have units of measure.

## Results

### Habitat Variables Driving Fish Assemblage Structure

In total 18,780 individual fish from 377 species were recorded from stereo-BRUVS. A Distance based Linear Model (DistLM) identified that depth explained the largest proportion of the variation in the fish assemblage (8.5%) (Figure 1, Table 1). Out of a possible 19 habitat variables, 13 comprise the optimum model explaining a cumulative total variation of 20.8%. These included % cover of rhodolith, hard coral, sponge, coralline algae, gorgonian and seawhip, branching coral, macroalgae, sand, turf algae, tabulate coral and seagrass (Figure 1, Table 1).

### Univariate Assemblage Structure

The results of the univariate Permutational Analysis of Variance (PERMANOVA) [31], [32], [33], [34] highlight a significant main effect of habitat for species richness, overall MaxN, Shannon diversity and trophic level (Table 2). This significant effect of habitat was driven primarily by the differences between 15–30 m and 30–50 m depth ranges (Table 2, Figure 2). The habitat with lowest overall species richness was sand at a depth of 50–70 m (3.1±1.6 S.E.) and the highest was reef slope at a depth of 10–30 m (36.1±2.1 S.E.). Shannon diversity followed a similar relationship with reef slope at a depth of 10–30 m being highest in Shannon diversity (2.6±0.13 S.E.) and sand at a depth of 50–70 m lowest (0.8±0.18 S.E.). Overall MaxN also decreased with habitats across the shelf with the highest being recorded on the reef slope at a depth of 10–30 m (151.7±27.6 S.E.) and the lowest sand at a depth of 90+ m (14.1±2.3 S.E.).

Conversely the average length increased significantly as you moved across the shelf into deeper water (Table 2, Figure 2). The habitats containing the smallest average size of fish (157±63 mm S.E.) were inshore patch coral reefs in 2–4 m depths while sand at a depth of 70–90 m contained the largest overall average fish size (465±19 mm S.E.). The overall trophic level of fishes increased across the shelf with patch coral reefs at a depth of 2–4 m being lowest (3.1±0.03 S.E.) and sand at a depth of 90+ m being the highest (4±0.05 S.E.). The number of unique species at deeper habitats including sand at a depth of 70–90 m and sponge at a depth of 90+ m was similar to shallow coral reef habitats.

**Figure 3:**
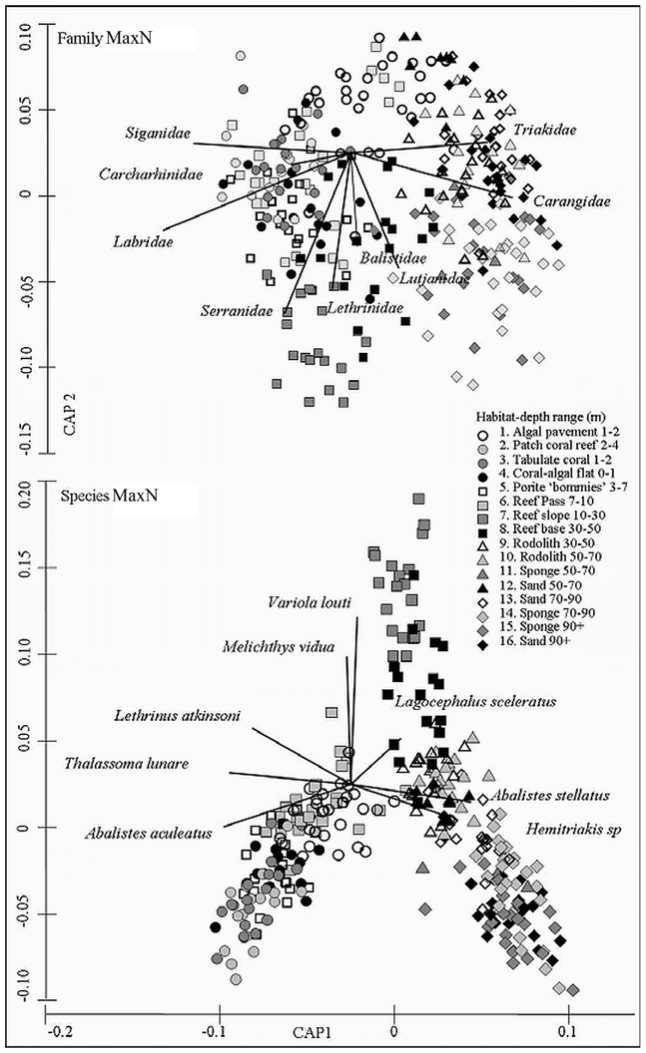
Family and species MaxN of fish in relation to 16 habitat zones. 62 species from 25 families have Pearson correlation values >0.25 and explain a majority of differences in fish assemblages between zones. A number of these species and families are represented on the respective plots with vectors illustrating the strength and direction of correlation to the 16 habitat categories.

**Figure 4:**
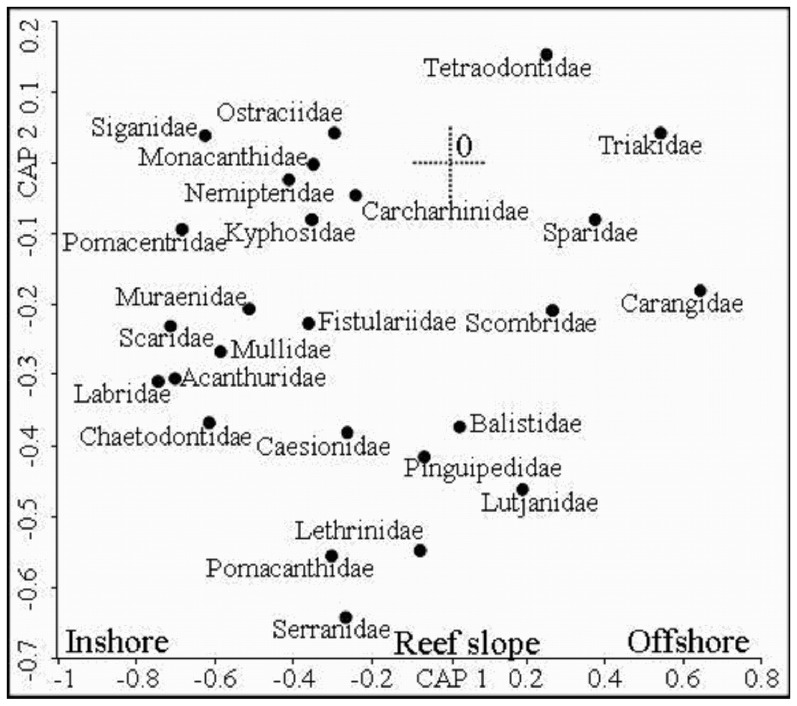
Showing 26 families with a Spearman rank correlation >0.25 from CAP plots with the exception of Carcharhinidae. Habitat affinities of the different families can be considered primarily related to sampling location either inshore or offshore.

**Figure 5:**
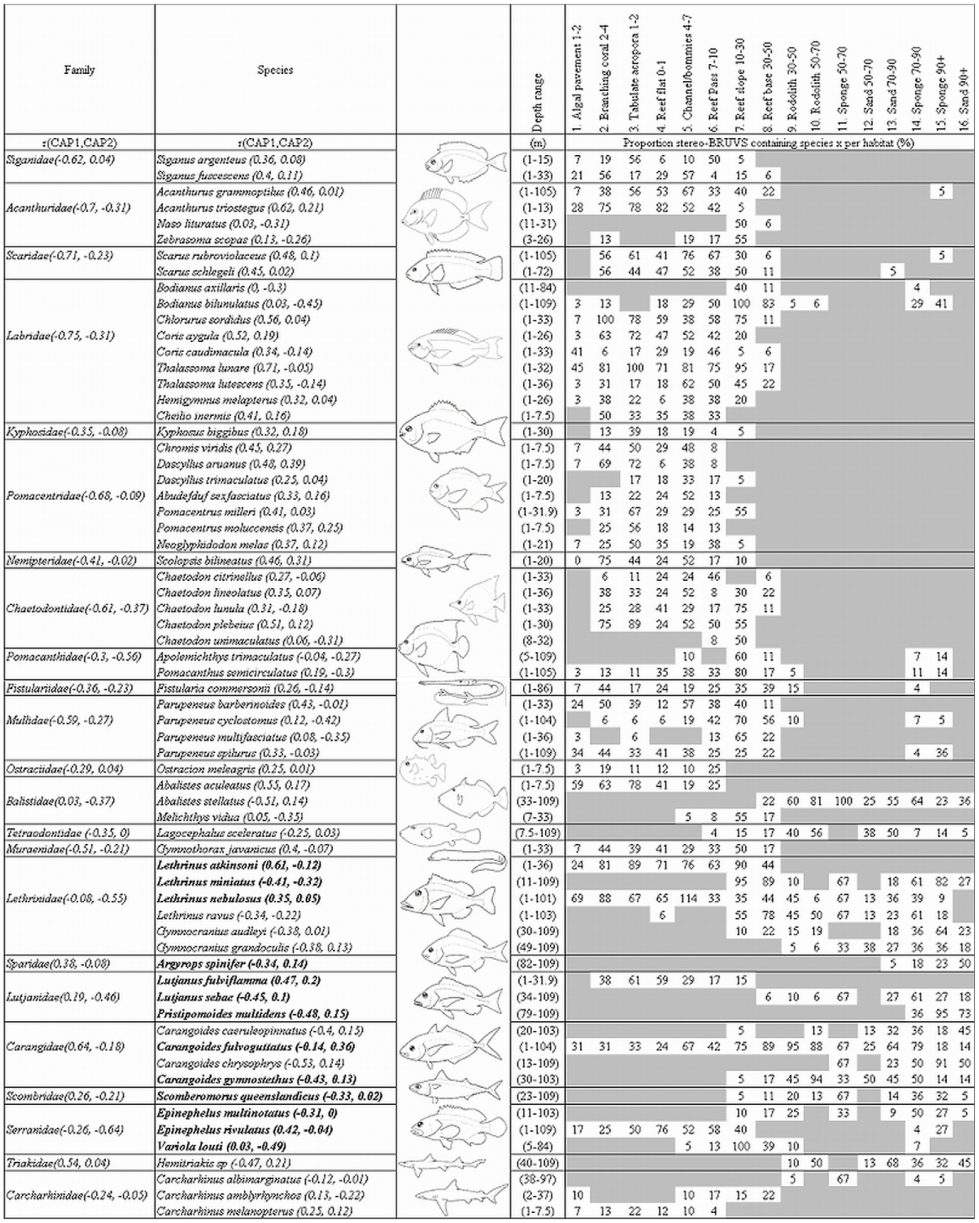
The proportion of stereo-BRUV stations within a particular habitat and depth combination which recorded the presence of a species. Family and species spearman rank correlations to CAP axis 1 & 2 indicated in brackets in addition to depth ranges.

### Multivariate Assemblage Structure

PERMANOVA revealed a significant main effect of habitat and depth for the species MaxN and family richness data (Table 2). In addition, species MaxN was the only measure for which a significant habitat/depth interaction was detected. Canonical Analysis of Principal Components (CAP) plots of this significant test were used to identify the groups driving these differences and illustrate the separation between samples from the 16 habitats across the shelf (Figure 3) [32], [35]. In both plots, fish assemblage data from inshore sites were proximal to each other as were those from the reef pass, slope and base and finally the offshore sites. Pair wise comparisons across both data sets revealed that out of the 120 possible comparisons only 3 were not statistically significant in both instances. PERMANOVA was used to test for significant differences between habitats for genus, order and class richness and all showed significant main effects of depth and habitat. Of the 120 pair wise comparisons between habitat categories, only 4 for genus, 14 for order and 20 for class comparisons were not significant (Table 2).

**Figure 6:**
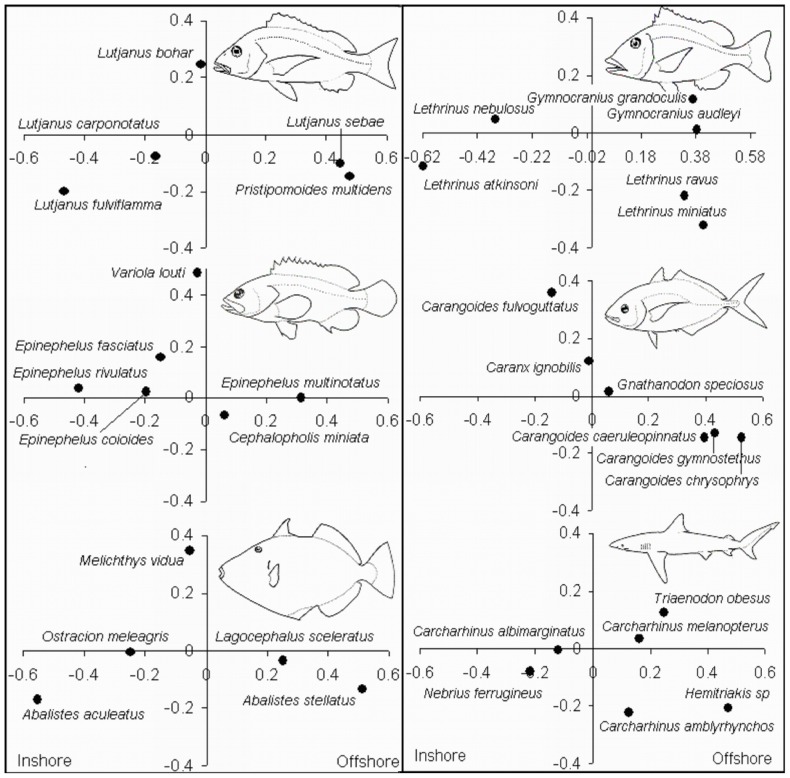
Showing species from families that have a Spearman rank correlation >0.25 from CAP plots. These plots demonstrate significant stratification of species across continental shelf habitat and depth gradients.

### Species and Family Trends

From fish assemblages across the shelf, 62 species from 25 families had a Pearson correlation value of greater than 0.25 (Figure 4, Figure 5). The leave one out allocation success from the CAP analysis [35] correctly identified a sample as belonging to one of the 16 habitat/depth categories 61% of the time suggesting fish assemblages were quite distinct.

**Figure 7:**
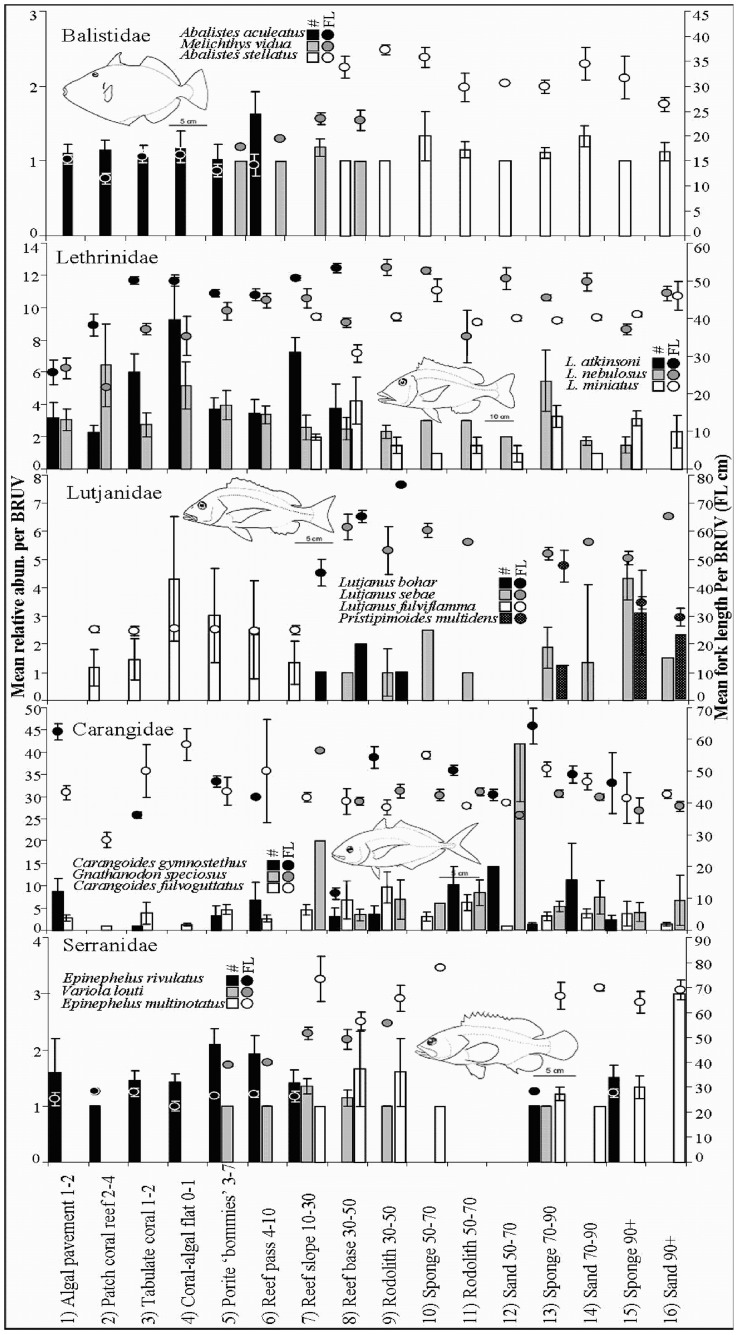
Average MaxN and length distributions across continental shelf habitats and depths for species of Balistidae, Lethrinidae, Lutjanidae, Carangidae and Serranidae. Significant habitat partitioning between conspecific species was evident with smaller bodied species inshore and larger bodied species offshore. Varying size distributions across habitat and depth gradients within species was indicative of ontogenetic habitat shifts e.g. Lethrinus nebulosus, L. atkinsoni and L. miniatus.

Fish families strongly associated with inshore habitats included *Chaetodontidae*, *Labridae*, *Acanthuridae*, *Mullidae*, *Scaridae*, *Muraenidae*, *Kyphosidae*, *Siganidae*, *Nemipteridae*, *Monacanthidae* and *Ostraciidae* (Figure 4). Families strongly associated with offshore habitats included *Tetraodontidae*, *Traikidae*, *Sparidae*, *Carangidae* and *Scombridae*. Families strongly associated with exposed reef slope, base and pass habitats included *Lutjanidae*, *Balistidae*, *Lethrinidae*, *Pomacanthidae*, *Serranidae*, *Pinguipedidae*, *Caesonidae*, *Fistularidae* and *Carcharhinidae*.

Generally, families strongly associated with reef slope to offshore habitats contain large bodied predatory species.

Within families, species of *Carangidae*, *Lutjanidae*, *Lethrinidae*, *Serranidae*, *Balistidae* and *Carcharhinidae* display varying degrees of habitat partitioning (Figure 6). In the case of *Serranids* for example *Epinephelus rivulatus*, *E. fasciatus* and *E. coioides* were most often associated with inshore lagoon habitats while *Variola louti* and *Cephalopholis miniata* were predominantly associated with the reef slope, base and pass habitats. By contrast *E. multinotatus* were most common on offshore habitats (Figure 6).

**Figure 8:**
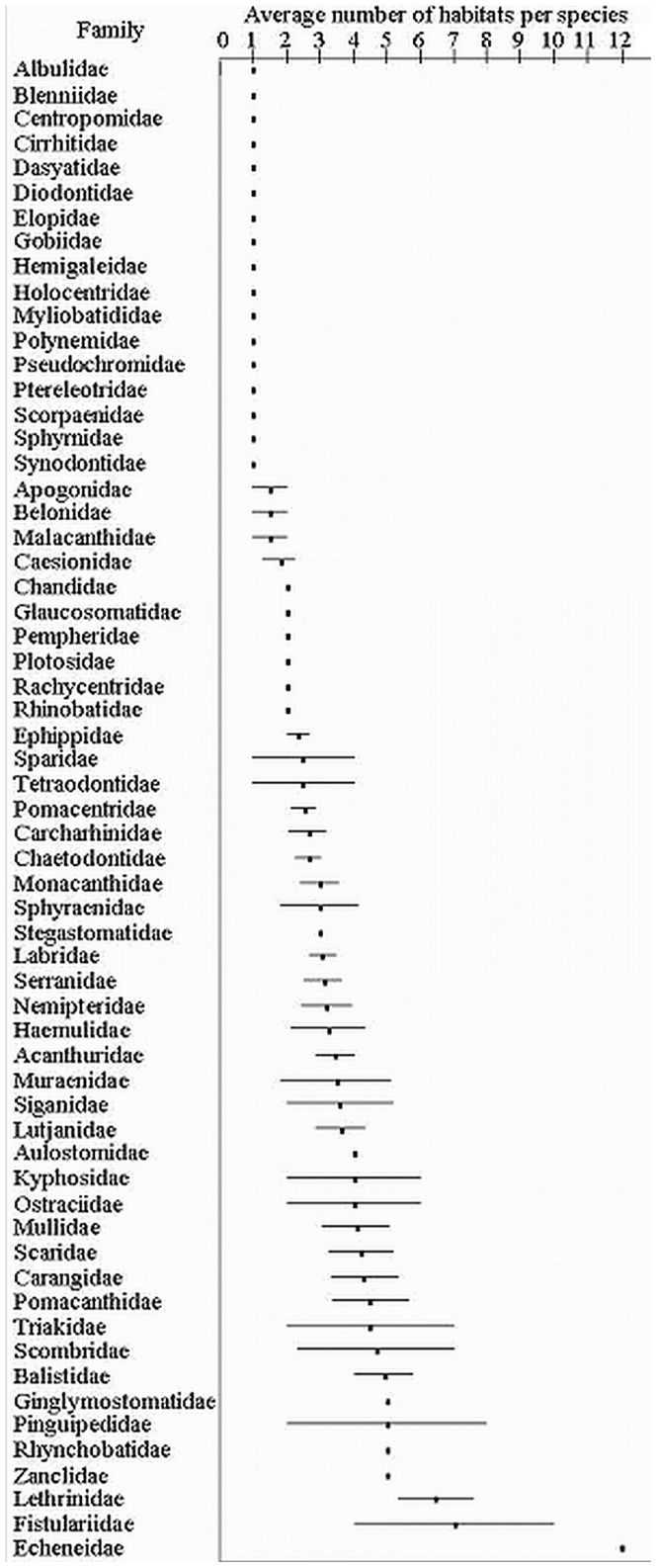
Relative comparison indicating degree of habitat specialization within demersal fish families. Average number of habitat/depth categories of species from the same family censused in this study are indicated by dots. Bars indicate min and max number of habitats for species from the same family.

**Table 3:** Species maximum depth recorded during this study compared to previous published records.

Family	Species	Max depth (m)		Family	Species	Max depth (m)	
		Previous	Current study			Previous	Current study
*Acanthuridae*	*Acanthurus grammoptilus*	20	105	*Lethrinidae*	*Lethrinus miniatus*	35	109
	*A. blochii*	15	80		*Gymnocranius audleyi*	40	109
	*A. mata*	25	80		*L. ravus*	35	103
	*Naso annulatus*	60	80		*L. nebulosus*	75	101
	*A. triostegus*	5	13		*L. laticaudis*	35	54
*Balistidae*	*Sufflamen chrysopterus*	30	97		*L. rubrioperculatus*	40	57
	*Balistoides viridescens*	50	101		*L. atkinsoni*	25	36
*Caesionidae*	*Pterocaesio marri*	30	36		*Gymnocranius grandoculis*	100	109
*Carangidae*	*Carangoides hedlandensis*	50	101	*Lutjanidae*	*Symphorus nematophorus*	50	84
	*C. chrysophrys*	60	109		*Lutjanus vitta*	72	100
	*C. gymnostethus*	70	103		*L. sebae*	100	109
	*C. ferdau*	60	92	*Mullidae*	*Parupeneus spilurus*	30	109
	*C. fulvoguttatus*	100	104		*P. barberinoides*	15	33
*Carcharhinidae*	*Carcharhinus albimarginatus*	20	97		*P. cyclostomus*	92	104
*Chaetodontidae*	*Coradion altivelis*	15	101	*Pinguipedidae*	*Parapercis nebulosa*	30	82
	*C. assarius*	40	102		*P. clathrata*	50	75
	*C. auriga*	35	86	*Pomacanthidae*	*Chaetodontoplus personifer*	30	103
	*Heniochus acuminatus*	75	109		*Pomacanthus semicirculatus*	40	105
	*C. plebeius*	10	30		*Apolemichthys trimaculatus*	60	109
	*C. speculum*	30	42		*P. imperator*	60	103
	*C. trifascialis*	12	24	*Pomacentridae*	*Pomacentrus milleri*	6	32
*Chanidae*	*Chanos chanos*	30	37		*Pomacentrus coelestis*	12	33
*Echeneidae*	*Echeneis naucrates*	50	99		*Neoglyphidodon melas*	12	21
	*Platax teira*	25	80	*Rachycentridae*	*Rachycentron canadus*	40	55
	*P. batavianus*	40	86	*Rhynchobatidae*	*Rhynchobatus djiddensis*	50	83
	*P. pinnatus*	25	34	*Scaridae*	*Scarus rubroviolaceus*	30	105
*Haemulidae*	*Diagramma pictum*	40	80		*S. ghobban*	30	103
*Kyphosidae*	*Kyphosus biggibus*	25	30		*S. frenatus*	25	84
*Labridae*	*Choerodon jordani*	40	109		*S. schlegeli*	50	72
	*Labroides dimidiatus*	40	86	*Scombridae*	*Scomberomorus commerson*	70	82
	*Cirrhilabrus punctatus*	32	60		*S. queenslandicus*	100	109
	*Choerodon cauteroma*	30	54	*Serranidae*	*Epinephelus multinotatus*	90	103
	*Coris pictoides*	30	42	*Siganidae*	*Siganus fuscescens*	4	33
	*Thalassoma lunare*	20	32	*Sphyraenidae*	*Sphyraena barracuda*	15	109
	*C. caudimacula*	25	33	*Stegastomatidae*	*Stegostoma fasciatum*	70	102
	*Hemigymnus fasciatus*	25	33	*Tetraodontidae*	*Lagocephalus sceleratus*	100	109
	*T. lutescens*	30	36				

(taken from: [36], [37]. Max. depth sampled this study 109 m.

### Habitat Specificity vs. Generality

There were 156 species restricted to one habitat-depth category and 231 species limited to 3 or less (Figure 5). Forty six species occurred only on the reef slope and generally inshore habitats supported more unique species than offshore. However, sponge dominated benthos at a depth of 70–90 m and 90+ m depth zones also supported high numbers of unique species (Figure 2). Many species from families commonly targeted by fishers were habitat generalists with relatively broad cross-shelf distributions including the *Lethrinidae* (7.62±1.14 S.E.), *Scombridae* (7±2.33 S.E.), *Carangidae* (5.31±0.99 S.E.), *Lutjanidae* (4.36±0.75 S.E.), *Serranidae* (3.66±0.56 S.E.) and *Carcharhinidae* (3.19±0.56 S.E.) (Figure 5, Figure 7 and 8). Only 13 species were found to frequent 10 or more habitats. *L. nebulosus* is the species most heavily targeted by fishers and was recorded in 15 habitats. *Carangoides fulvoguttatus* was the most abundant *Carangid* and was recorded in all 16 habitats. Species of *Scaridae* and *Pomacanthidae* averaged the same broad habitat distributions as *Carangidae* while species from other families were similarly broadly distributed (Figure 8).

### Species Lengths

Average fish length increased across shelf depths and habitat. This reflected the decreasing occurrence of small bodied shallow water habitat specialists including *Siganidae*, *Acanthuridae*, *Scaridae*, *Labridae*, *Pomacentridae*, *Chaetodontidae* and *Mullidae* families (Figure 5). Additionally, the *Lethrinidae*, *Lutjanidae*, *Carangidae*, *Balistidae* and *Serranidae* display increasing average length across the shelf. This reflected varying levels of ontogenetic habitat partitioning within the species and the partitioning of habitat between different species. Larger bodied individuals and larger bodied species were generally associated with offshore habitats (Figure 8).

**Figure 9:**
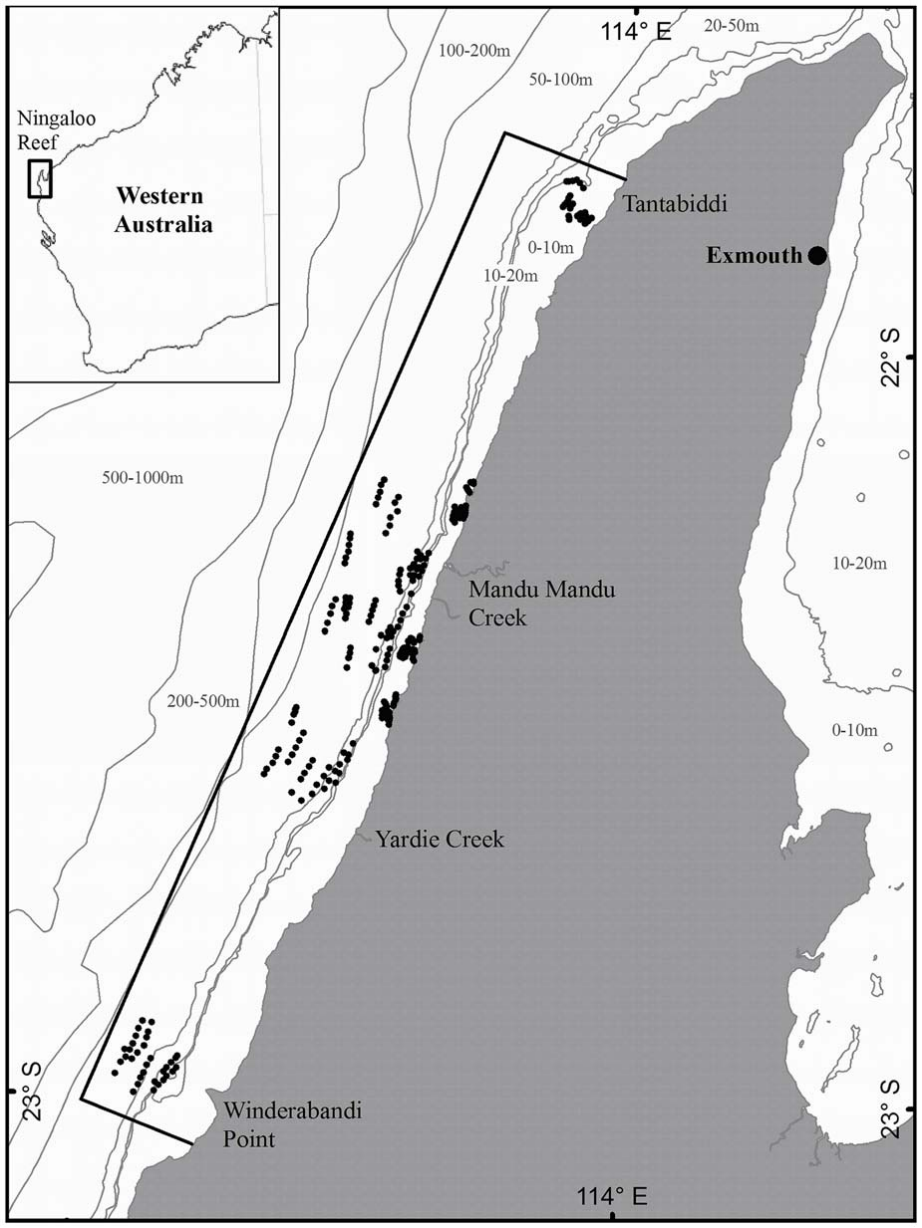
Map of Western Australia showing the location of Ningaloo Reef. The northern Ningaloo Reef and adjacent bathymetric contours expanded with the perimeter of the study site bounded by the box extending from Winderabandi point in the south to Tantabiddi in the north. The reef crest shown demarking between inshore and offshore waters.

### Depth Range Extensions

Many species were sampled at depths beyond their maximum records (taken from [36], [37]). These included species of Acanthuridae (Naso tuberosus, Acanthurus mata, A. blochii, Naso annulatus, A. grammoptilus), Balistidae (Sufflamen chrysopterus, Balistoides viridescens), Chaetodontidae (Chaetodon auriga, Coradion altivelis, Chaetodon assarius, Heniochus acuminatus), Labridae (Labroides dimidiatus, Choerodon jordani), and Scaridae (Scarus schlegeli, S. frenatus, S. ghobban, S. rubroviolaceus) (Table 3).

## Discussion

The influence of habitat and depth on fish assemblage structure was evident across the continental shelf at Ningaloo Reef. Generally fish assemblages inshore differed from those on the reef pass, reef slope and reef base habitats and also offshore sites dominated by rhodolith and sponge/soft coral communities. Depth was the most significant factor in explaining these differences, however other variables which were generally indicative of major shifts in benthic habitat type such as rhodoliths, hard coral and sponge cover in combination with depth accounted for 20% of the variation in fish assemblages.

### Assemblage Level Patterns

Overall univariate assemblage level patterns included a decline in species richness, average overall MaxN and Shannon diversity across the shelf. In contrast average overall lengths increased significantly offshore. Fish lengths inshore averaged between 200 and 300 mm while offshore average length was around 400 mm. Offshore habitats had less species, but supported greater MaxNs of higher order predators, which was likely to have significant implications for overall assemblage structure based on the relative importance of predation [38].

**Table 4:** Depth/habitat factor groups and number of stereo-BRUVS replicates sampled.

Habitat/Depth	Total
1) Algal pavement 1–2	29
2) Patch coral reef 2–4	16
3) Tabulate coral 1–2	18
4) Coral-algal flat 0–1	17
5) Porite ‘bommies’ 3–7	21
6) Reef pass 4–10	24
7) Reef slope 10–30	20
8) Reef base 30–50	18
9) Rhodolith 30–50	20
10) Sponge 50–70	16
11) Rhodolith 50–70	3
12) Sand 50–70	8
13) Sponge 70–90	22
14) Sand 70–90	28
15) Sponge 90+	22
16) Sand 90+	22

### Unique and Rare Species

The number of unique species specific to one of the habitat/depth combination, as well as richness at the level of Genus, Family, Order and Class also vary significantly with habitat and depth. These patterns contribute to the ability to discern consistent differences between fish assemblages even at lower taxonomic levels [39]. Habitats with highest species richness and most unique fish generally occur in 30 m or less of water, however offshore sponge habitats were also high in unique species from a number of families including some not represented in shallower waters such as *Carcharhinus albimarginatus, Epinephelis multinotatus, Pristipomoides multidens, Lutjanus sebae, Carangoides chrysophrys, Argyrops spinifer,Gymnocranius grandoculis and Abalistes stellatus* and *Sparidae* and *Triakidae* families. There were 156 species restricted to only one habitat-depth category and 231 species limited to 3 or less. Offshore deeper water sponge-dominated habitats contained similar numbers of unique species as shallow water coral reefs, suggesting similar susceptibility to habitat specific impacts [40].

### Shallow Water Impacts

Overall it was clear that shallow water pressures will affect the highest numbers of habitat specialists, with the majority of such species entire post recruitment populations closely associated with shallow coral reef habitats [1]. Broadscale shallow water impacts may also affect the MaxN of particular species that contribute to the structure of fish assemblages across the continental shelf. Although the majority of species were restricted to three or less habitats within 30 m water depths, many families with highly specialized species also had closely related fishes with extended depth ranges well below those previously recorded before, including abundant coral reef fish from the families *Acanthuridae*, *Chaetodontidae*, *Labridae*, *Pomacanthidae*, *Pomacentridae* and *Scaridae*.

### Habitat Generalists

Species which occupy a large range of habitats will be more resilient to disturbance than habitat specific species. This will have significant implications for their population and conservation biology [41]. The response to shallow water disturbances of species that utilize broader depth ranges will be dictated largely by their physiology. Factors such as tropical species being restricted by colder temperatures at depth may lead to reduced growth rates, increasing size at sexual maturity, reduced fecundity and reduced contribution of recruits [42]. Many species including *Bodianus bilunulatus*, *Pomacanthus sexstriatus* and *Heniochus acuminatus,* commonly observed by divers on adjacent shallow reef, were found in moderate MaxNs in depths of up to 100 m. Understanding the implications of shallow water impacts for population maintenance of a range of species will require dedicated biological and demographic studies on populations that exist at depths >30 m. Even closely related species were associated with highly contrasting ranges of habitats, demonstrating their response to habitat-specific impacts will differ.

### Trophic level, Size and Habitat Partitioning

Increasing length with depth was consistent with ontogenetic habitat shifts in many members of the *Balistidae*, *Lethrinidae*, *Lutjanidae*, *Carangidae* and *Serranidae* families. Small size classes of *Lethrinidae* species in particular can be abundant in specific habitats, while larger size classes can be found at lower densities utilizing a broader range of habitats [43]. This pattern should be treated with caution, as size selective mortality due to the effects of fishing on larger individuals in shallow habitats has been shown at this location [44]. At the family level, habitat partitioning between species from the same family contributes to the observed patterns of increased average length with depth, resulting in smaller species inshore and larger species offshore. This abrupt partitioning of habitat between species was found in *Balistidae*, *Lethrinidae*, *Lutjanidae*, *Carangidae* and *Serranidae* fish guilds and supports the view that competitive interactions are an important process structuring fish assemblages [45]. Offshore habitats were composed almost entirely of these families and their abundances contributed most to the differences between the observed fish assemblages. This has implications for which ecological processes contribute most to fish assemblage structure across these habitat and depth gradients. It also suggests that higher order predators, which as adults frequent a range of habitats, as well as generalist species from lower trophic levels, will respond differently to impacts on shallow water food and shelter resources.

### Conclusions

The effectiveness of shallow water no-take zones for providing protection to these more generalist target species from fishing impacts was likely to depend upon extent of ontogenetic shifts away from shallow habitats [46]. Target fishes whose abundance has been depleted in shallower waters contribute most of the MaxN in less speciose fish assemblages at offshore habitat [44]. Globally no-take zoning is predominantly applied to shallow water habitats, with a minority extending across adjacent continental shelves. Various non-target species were found to utilize a range of habitats whilst many more were highly specific in their habitat associations. Species with isolated populations found at different depths may represent discrete populations that function independently of one another and ecosystems processes maintaining them may differ [21]. The spatial extent and relative magnitude of critical ecosystem processes contributed by various species are also likely to be orders of magnitude different. These factors suggest dedicated ecological and demographic studies of fish assemblages across continental shelves are important in defining the habitat needs of fish assemblages and identifying possible management implications [8]. Human impacts such as climate change, fishing and pollution will have profoundly different ecological implications depending upon where they occur [47]. To be effective, fisheries management and marine conservation agencies need to incorporate the full range of continental shelf habitats that demersal fish utilize, into their management plans.

## Materials and Methods

### Ethics Statement

This work was conducted under University of Western Australia Animal Ethics Approval RA/3/100/529 which adheres to Federal Australian Government Code of Practice. This work complied with all relevant government regulations including Department of Environment and Conservation’ Authority to enter CALM land and/or waters permit number CE001708 and license to take fauna for scientific purposes SF005913; and given an exemption to Fisheries Western Australia Fish Resources Management Act 1994 by the Director of Fisheries Research.

### Study Site

Ningaloo Reef is a fringing tropical coral reef approximately 300 km long and lies adjacent to the semi-arid North West Cape of Western Australia between 23° 48.00'S and 21° 48.00'S. The entire fringing reef system and adjacent shelf waters are declared a Marine Park. This study was undertaken within the shallow continental shelf waters <110 m of northern Ningaloo region (Figure 9). This northern section of reef has a lagoon <5 km wide and is less than 10 m deep. The reef is punctuated by regular passes which are deep channels that funnel water from the lagoon to the open ocean and has a steep fore reef slope down to approximately 30 m, before sloping gently across a narrow continental shelf to the shelf break <5 nautical miles seaward of the reef crest [48].

### Defining Broadscale Habitats

Pre-existing benthic habitat maps were available for the study areas. Habitat maps were derived from different sources depending on whether they were inshore or offshore. Maps of the geomorphology and associated modern habitats in shallow water inshore <20 m were used to locate sites in 6 inshore habitat/depth categories [49], [50]. These included algal pavement 1–2 m which are areas of exposed limestone platforms near shore and colonized by diverse macroalgae communities, patch coral reef 2–4 m which are areas of isolated coral dominated reef surrounded by sand, tabulate coral 1–2 m which are substantial back reef areas dominated by tabulate colonies, coral-algal flat 0–1 m which are reef flats dominated by coralline algae and rubble, porite 'bommies' 3–7 m which are large colonies of massive porites supporting extensive coral reef growth and the reef pass 4–10 m which are deep channels that funnel water from the lagoon to the open ocean (Table 4).

Maps of the geomorphology and associated modern habitats in deep water offshore outside the reef crest <15 m and >110 m were used to locate sites in 10 offshore habitat/depth categories [51]. These included Reef slope 10–30 which are the steeply sloping reef front, Reef base 30–50 which are composed of broken bottom, rubble and sand at the base of the reef slope, Rhodolith 30–50 which are areas of habitat dominated by extensive beds of rhodoliths, Sponge 50–70 which are reef and substrate dominated by filter feeding sponges, Rhodolith 50–70, Sand 50–70, Sponge 70–90, Sand 70–90, Sponge 90+ and Sand 90+ categories (Table 4). The classification of these categories enabled us to plan the stratification of our finescale habitat and fish sampling.

### Finescale Benthic Habitat Sampling

Finescale habitat sampling was different depending on whether sampling was inshore or offshore. At four areas, finescale habitat sampling was undertaken within each of the six inshore habitat/depth categories (Figure 9). Scuba divers recorded benthic habitat with a video camera held ∼30 cm above the substratum along five random 50 m transects [52], [53]. 20 stills taken randomly from each video transect were then censured using a point sampling technique in which 10 randomly dispersed points were sampled per image resulting in a total of 200 randomly defined points sampled per transect [52], [53]. The points were classified as belonging to various physical and biological variables including % cover massive coral, submassive coral, branching coral, tabulate coral, encrusting coral, foliose coral, digitate coral, macroalgae, turf algae, coralline algae, rhodoliths, soft coral, gorgonian, seawhips, sponge, seagrass, sand, overall hard coral and depth. Number of points were summed for each category and converted to provide an estimate of percent cover of major benthic categories.

At four areas, finescale habitat sampling was undertaken within each of the ten offshore habitat/depth categories (Figure 9)., A towed video system recorded benthic habitat from approximately 60 cm above the substratum along five random 100 m transects. The towed camera had a wide angle lens of 127° faced slightly forward at an angle of approximately 15 degrees and lighting was provided by two 6 watt dive torches. The resulting footage was analyzed as described for shallow transects above. Subsequently a multivariate matrix was compiled that quantified habitat variability stratified by the sixteen habitat/depth categories.

### Fish Community Sampling

Non-destructive baited remote underwater stereo-video systems (stereo BRUVS) were deployed to collect data on the abundance, assemblage composition and lengths of demersal fishes. Up to six random replicate stereo BRUV samples stratified by the same 16 habitat/depth categories sampled for finescale benthic habitat at four cross-shelf areas were deployed. A total of 304 samples were collected between April 2006 and July 2006 (Table 4, Figure 9). The use of stereo BRUVS allowed the standardization of the area sampled to account for differences in visibility between camera drops by controlling for the range at which fish were included in the samples [25], [54], [55]. The sampling area was standardized to 37.22 m^2^ by excluding fish that were beyond the minimum horizontal visibility of 6 m recorded across all stereo-BRUV drops [55]. This allowed us to make estimates of abundance and length of fishes in a consistent manner at stations across the shelf [56], [57].

The stereo-BRUVs deployed used paired Sony HC15 digital camcorders within waterproof housings. Bait arms made of 20 mm plastic conduit with a standard rock lobster bait canister fastened to one end of the frame. Also attached to this conduit was a diode in the field of view of both cameras, to enable synchronization of video frames for stereo measurements (see [28] for a full description). Approximately 800 gms of crushed *Sardinops sagax* or sardines were placed in the bait bag for each deployment. Six stereo-BRUVS were loaded with 2×1 hour video tapes, set to record and deployed simultaneously within a single habitat. To minimize the effect of bait odour on adjacent samples they were deployed at a nominal spacing of 250 m apart [58]. At deep or turbid sites where available light was likely to be low at the seafloor the stereo-BRUVS were set to record on ‘nightshot’. The stereo-BRUVs were retrieved after recording for one hour at each station then prepared and bait replenished for redeployment at another site.

### Image Analysis

Each stereo-BRUVS tape were assessed for the appearance of fish using the custom interface BRUVS1.5.mdb© developed by the Australian Institute of Marine Science (2006). The data base enabled the tape reader to record the video frame number the maximum number of individuals of the same species seen together on the whole tape occurred (MaxN). The use of MaxN as an estimator of abundance has been reviewed in detail by [13] and [59]. Estimates of MaxN are considered conservative, particularly in areas where fish occur in high densities. In the laboratory each stereo video pair was captured as a digital AVI file (audio video interleaved file) and compressed with DivX to reduce the overall file size. Stereo AVI pairs were synchronized and calibrated. Calibration files were derived using CAL 1.11 software following the procedure detailed in [22], [23]. The stereo-photo comparator PhotoMeasure (www.seagis.com.au) was used to measure the lengths of fish from the stereo video imagery.

### Data Analysis

#### Habitat variables correlated to fish assemblage structure

Distance based linear models (DistLM) were used to model the percent overall variation in fish assemblages accounted for by finescale variation in habitat and depth across the continental shelf [31], [60], [61]. The variation in habitat was assessed from the towed and diver video transects. DistLM used Pearson correlation R-values to identify main species and habitat variables explaining significant amounts of variation in fish assemblage data. The DistLM was based on a permutational procedure in which species MaxN data were converted into a modified Gower log 10 distance matrix then compared to the habitat data matrix using a forward stepping procedure which optimizes selection of variables explaining most variation in the fish assemblage. The direction and magnitude of the relationship between habitat variables and individual fish species were displayed using distance based redundancy analysis (dbRDA) biplots [61].

#### Univariate parameters

Statistical differences in univariate species richness and diversity, overall MaxN, length and trophic level were tested between different depth and habitat combinations. Overall MaxN is calculated as the sum of MaxN of all fish species per replicate, species richness is a count of the total number of different species viewed on the video and species diversity is calculated using the Shannon diversity index substituting MaxN instead of absolute abundance to calculate the index [62]. Number of unique species per habitat was considered and was the sum of unique species recorded from stereo-BRUV replicates within specific habitat/depth combinations. Values for trophic level for each species was obtained from fish base and where a value for a particular species was not available that of a morphologically similar and closely related species was substituted. Because normality in such data was not a reasonable assumption due to the predominance of zeros and the variability amongst habitats and depth zones, a two-way permutational analysis of variance was used (PERMANOVA) [31], [32], [33], [34]. For each term in the analysis, 4999 permutations based on Euclidean distance with no transformation were computed to obtain P- values [34]. Where significant main effects or interactions were detected, pair-wise comparisons between different depth and habitat combinations were undertaken to investigate where the differences were occurring.

#### Assemblage structure

Species and family MaxN and the number of species from a genus, family, order and class sampled between different depths and habitats were also compared using PERMANOVA. The use of MaxN for analyzing stereo-BRUVS video tapes results in conservative estimates of the relative abundance of fish [15]. This is because MaxN is only a count of the maximum number of individuals of a species seen at one moment on the footage and not every individual that might enter into the field of view of the camera during the entire replicate. A Modified Gower Logbase 10 dissimilarity measure was used to analyze the final MaxN data sets and the various measures of taxonomic richness since this measure places more emphasis on compositional change of the assemblage and less on changes in MaxN [63]. Family MaxN is a multivariate measure of the species MaxN summed up to the family level and is independent of when these species MaxN values were recorded on the video tape. Records of schooling fish species that appeared in high numbers (100 –1000 s) on individual stereo-BRUV samples, but were seen rarely on other samples were omitted as well as unidentified species data with the exception of the common *Hemitraikis* elasmobranch shark species. These data were analyzed using the model described above (4999 permutations).

Where significant differences in species and family MaxN were detected, plots of the principal coordinates were constructed from a constrained Canonical Analysis of Principal Coordinates (CAP) [32], [35]. This procedure maximizes separation between significant factors of depth and habitat and uses Spearman Rank correlations to identify which species and family groups contribute towards this significant difference. Since stereo-BRUVs sample a large cross-section of the fish assemblages with orders of magnitude differences in MaxN between different species, a Spearman correlation R value >0.25 was used to identify those families and species that were driving significant patterns between the factors. Specific family and species groups that had significant r-values were plotted on a separate set of axes to aid identification of significant habitat partitioning at family and species levels.
